# A protocol for a systematic review and meta-analysis of the diagnostic accuracy of mid-regional pro-adrenomedullin in predicting invasive bacterial infection in children

**DOI:** 10.1186/s13643-020-01338-1

**Published:** 2020-04-02

**Authors:** Michael Corr, Thomas Waterfield, Derek Fairley, James McKenna, Michael D. Shields

**Affiliations:** 1 Centre for Experimental Medicine, Wellcome Wolfson Institute of Experimental Medicine, Queen’s University Belfast, 97 Lisburn Road, Belfast, BT9 7AE UK; 2 Belfast Health & Social Care Trust, Belfast, UK

**Keywords:** Mid-regional pro-adrenomedullin, Adrenomedullin, Invasive bacterial infection, Sepsis, Diagnostic accuracy, Meta-analysis

## Abstract

**Background:**

The early recognition of invasive bacterial infections (IBI) in children can be difficult. Clinically it is often challenging to differentiate between the early stages of an IBI and a benign self-limiting viral infection. These challenges mandate a cautious approach resulting in the overuse of antimicrobial drugs with resultant antimicrobial resistance. Due to these challenges, there is growing research into the role of biomarkers for the early identification of children with IBI. Earlier and more accurate diagnoses may lead to improved clinical outcomes for children and reduced antimicrobial resistance. Mid-regional pro-adrenomedullin (MR-proADM) is a biomarker that has been shown to be elevated in patients with IBI. The aim of this systematic review is to determine the diagnostic accuracy of MR-proADM at identifying children with IBI.

**Methods:**

To identify relevant studies we will search MEDLINE, Embase, Web of Science and Scopus from 1980 to the present day for all human clinical trials involving children that report the test accuracy of MR-proADM. We will include case-control studies, cohort studies and randomised control trials reported in any language. In addition, we will hand-search reference lists and grey literature including conference abstracts and web searches. Two reviewers will independently screen study titles and abstracts for eligibility followed by full-text assessment and data extraction including population, setting, timing and use of index test and reference standard used. Methodological quality will be assessed, by two authors, according to the revised tool for the quality assessment of diagnostic accuracy studies (QUADAS-2), any discrepancies will be resolved by a third author. The following test characteristics will be extracted into 2 × 2 tables for all included studies: true positives, false positives, true negatives and false negatives. Study-specific estimates of sensitivity and specificity with 95% confidence intervals will be displayed in forest plots.

**Discussion:**

This review will report the normal ranges for MR-proADM in health and the diagnostic accuracy of MR-proADM at identifying children with IBI. The review will help to define where in the diagnostic pathway MR-proADM could be useful including potential as a point-of-care test for children at first presentation with IBI.

**Systematic review registration:**

PROSPERO CRD42018096295

## Background

### Target condition being diagnosed

Invasive bacterial infections (IBI) are defined as the identification of pathogenic bacteria from a sterile fluid or body tissue by either culture or polymerase chain reaction (PCR) techniques [1]. Invasive bacterial infections are a major cause of morbidity and mortality in the paediatric population [2]. The United Nations Children’s Fund (UNICEF) estimates that the majority of mortality in under-fives globally is a result of invasive infections, particularly in low-income countries. They also estimate that one in three neonatal deaths globally is a result of IBI, specifically sepsis and meningitis [3, 4].

**Fig. 1:**
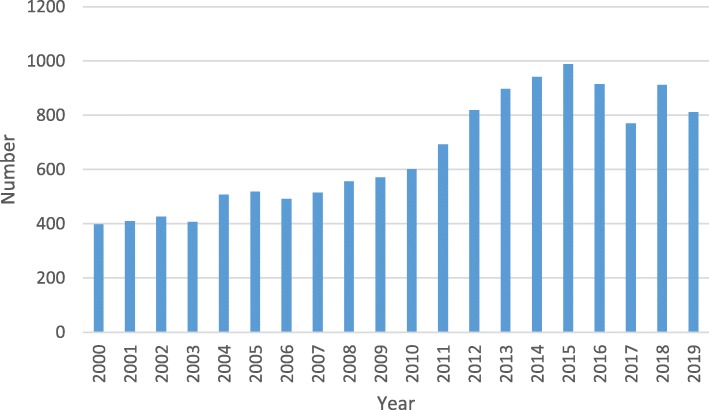
Number of articles registered with PubMed per year for biomarkers and infections. There has been an overall increase in the number of articles published on infective biomarkers. Data was taken from https://www.ncbi.nlm.nih.gov/pubmed/ - Accessed 26/02/2020.

Clinically it is challenging to identify those children with an evolving IBI from those with a simple self-limiting viral infection due to the non-specificity of initial presenting symptoms (particularly amongst those of the youngest age) [5]. This has led to increasing research (Fig. 1) into accurate and rapid diagnostic testing for biomarkers of IBI, increasing the need for systematic reviews of these novel markers as evidence becomes available.

### Index test and alternative tests

A growing number of biomarkers are being described and researched including C-reactive protein (CRP), Procalcitonin (PCT), cytokines such as interleukin 6 (IL-6) and adrenomedullin (specifically mid-regional pro-adrenomedullin). As the body of evidence grows, there have been an increasing number of reviews, including systematic reviews published [6, 7]. Unfortunately, neither CRP nor PCT is ideal biomarker for detecting early IBI due to their poor specificity and delay in rise from the onset of symptoms [6, 7]. Of the cytokines being investigated as potential biomarkers for IBI, the cytokine IL-6 has shown promise and there is an ongoing Cochrane Review exploring its role in sepsis in adults [8].

The index tests for this review are mid-regional pro-adrenomedullin (MR-proADM). MR-proADM is related to the peptide adrenomedullin (ADM) which was first discovered in 1993 and found to have potent and long-lasting vasodilatory effects [9]. ADM has been shown to be involved in a wide range of physiological processes in normal human biology and is regarded as a circulating hormone with significant paracrine and biological activities; the normal range for ADM in healthy individuals is 2 to 4 pmol/l [10]. ADM levels have been shown to rise early in serious infection and specifically in response to hypoxia and the production of cytokines such as interleukin-1, IFY-ϒ and tumour necrosis factor within a similar timeframe to other commercially used infective biomarkers [11–14]. ADM levels do not appear to rise to the same extent in mild self-limiting viral infections and the levels of ADM reported to correlate with disease severity [15–18].

Adrenomedullin is however difficult to measure owing to its instability as a molecule, rapid binding to receptors, fast metabolism and short half-life [18]. Mid-regional pro-adrenomedullin (MR-proADM) is a 48 amino acid fragment of ADM produced during ADM synthesis at a ratio of 1:1. Although biologically inactive, its longer half-life and relative stability makes measurement from body fluids easy and reliable [15]. These features make MR-proADM an exciting prospect as a new biomarker for IBI in children.

Unfortunately, most of the research conducted so far has been in adult populations with a systematic review of the paediatric literature required to understand the value of MR-proADM in assessing children with possible IBI including data relating to normal ranges in health and ranges in disease.

### Clinical pathway

**Fig. 2:**
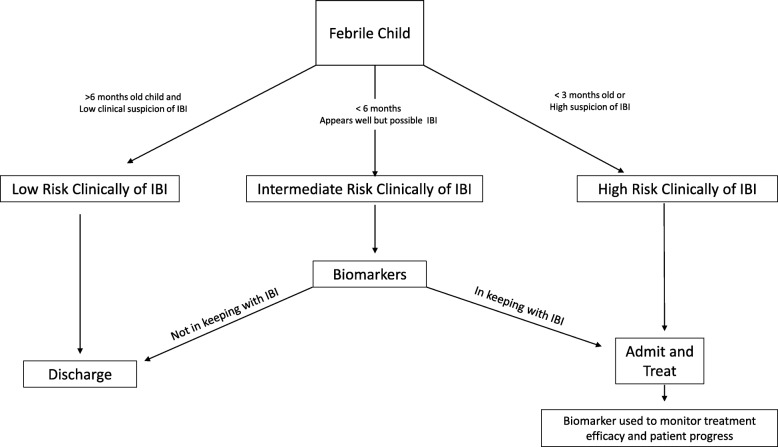
Clinical pathway of a child presenting with fever

Diagnosis of early IBI in children represents a significant clinical challenge. Fever is the leading reason for a child to be brought to the emergency department [19]. Most will have a self-limiting viral infection [7], but a small proportion will have an underling life-threatening IBI [7]. Often the diagnosis is not immediately obvious, and children undergo additional testing including for biomarkers of infection [19]. These biomarkers are then used to help determine (i) if antibiotic treatment is required and (ii) to monitor response to antibiotic treatment (iii) prevent the overuse of antibiotics in those children who have a self-limiting viral infection. The existing biomarkers in clinical use are poorly accurate for this purpose leading to some children receiving unnecessary treatments whilst others are falsely reassured. A new biomarker with superior accuracy to existing biomarkers could be used to decide the appropriate treatment for children with signs of possible IBI and to better monitor those on treatment. Fig. 2 outlines a simplified summary of the approach to suspected infection in children and shows where biomarkers may be used in the clinical pathway.

If MR-proADM demonstrates a high diagnostic accuracy for identifying children with an early IBI then it could be used to help better identify those children requiring immediate treatment from those who could be safely managed in the community or in fact not require antimicrobial therapy. Reducing the use of parenteral antibiotics has the potential to reduce antimicrobial resistance.

Furthermore, serial measurements of MR-proADM could be used to monitor the response to treatment or predict those requiring intensive care.

### Rationale

There is a need for a reliable and accurate biomarker to identify those children with an early IBI from those with a self-limiting viral infection. MR-proADM has been shown to be a stable and reliably measurable peptide with many of the properties of an ideal biomarker. The purpose of this systematic review is to synthesise and critically appraise all of the available research reporting on the diagnostic accuracy of MR-proADM in identifying children with IBI [17]. Data from adult studies suggest that a MR-proADM level of above 0.8 nmol/l is indicative of possible IBI [20].

### Objective

The primary objective of this systematic review is to determine the diagnostic accuracy of MR-proADM at detecting IBI in children under 18 years of age. The secondary objective is to determine, via subgroup analyses, whether the diagnostic accuracy of MR-proADM differs between newborns, neonates, infants, children and adolescents and if different optimal cutoff values exist between different age groups. These groups have been chosen to reflect the differing physiological and host responses to infection between age groups.

## Methods and design

We will perform a systematic search for all relevant studies; afterwards we will screen and select suitable studies for inclusion against our eligibility criteria. Data extraction will be performed in duplicate on selected studies in order to complete a meta-analysis.

The Preferred Reporting Items for Systematic Reviews and Meta-analysis Diagnostic Test Accuracy (PRISMA-DTA) standards will be adhered to when we are reporting the findings of this review [21]. The content of this protocol follows Preferred Reporting Items for Systematic reviews and Meta-analysis Protocols’ (PRISMA-P) recommendations [22]. (Please see attached the additional file 1 for the completed PRISMA-P checklist). This review is registered with the International Prospective Register of Systematic Reviews (PROSPERO) [23]. The registration number is CRD42018096295.

### Inclusion criteria

#### Types of studies

All case-control studies, cohort studies and randomised control trials reported in any language that assess the performance of MR-proADM in assessing children (< 18 years of age) for potential IBI will be considered. Case control studies will be included in the systematic review to aid with the description of normal ranges in health and disease but will be excluded from any meta-analysis. Only diagnostic test accuracy studies adherent the STARD (Standards of Reporting Diagnostic Accuracy Studies) [24] criteria will be eligible for inclusion in the meta-analysis.

#### Participants

Children (< 18 years of age) with clinician suspected infection undergoing MR-proADM testing.

(Table 1 Summary of inclusion criteria for this review)

**Table 1:** Inclusion criteria

Study characteristics	Inclusion criteria
Population	Children < 18 years of age with suspected invasive bacterial infection
Index tests	Mid-regional proadrenomedullin
Reference test	Quantitative PCR and/or culture of sterile site (blood, urine and/or CSF) specimens
Outcomes	True and false positives, true and false negatives
Study designs	All prospective, retrospective and randomised control studies that report measures of diagnostic accuracy of mid-regional pro-adrenomedullin in the diagnosis of IBI.

#### Index tests

The index test being measured is MR-proADM. Index testing can be performed on any bodily fluid (including but not limited to blood/urine/cerebrospinal fluid) using commercially and non-commercially available tests.

#### Target conditions

Invasive bacterial infections (IBI) defined as infections of sterile sites such as blood and cerebrospinal fluid (CSF).

#### Reference standards

The reference standard used to confirm the presence of the target condition in this study will either be positive blood or cerebrospinal fluid culture or PCR for pathogenic bacterial infection taken at the same time as the index text. No other reference standard will be accepted. These reference standards are widely accepted as the gold standard for diagnosing IBI [25, 26].

### Exclusion criteria

There is little paediatric experience of using MR-proADM and as such all eligible studies will be considered and included in the systematic review. Only studies that adhere to STARD criteria for reporting diagnostic test accuracy will be included in any meta-analysis. This approach will allow for a transparent summary of the current literature and reporting of normal ranges in health whilst also allowing for meta-analysis of robustly designed diagnostic accuracy studies.

### Search methods for identification of studies

#### Electronic search strategy

In collaboration with the Queen’s University Belfast Medical Librarian (RF), an electronic search strategy has been developed. We will search MEDLINE, Embase, Web of Science and Scopus. An example of Medline search strategy is attached in Supplementary File 2. There are no language restrictions for this review. Studies written in languages that authors are not fluent in will be translated using the official translation services at Queen’s University Belfast. There will be no time restriction on articles to be included in this review.

#### Searching other sources

In addition to our electronic search strategy, we will hand-search referenced lists of relevant articles. A targeted grey literature search will also be conducted by review of clinical trials databases, conference abstracts, internet searches and review articles. Mendeley electronic reference manager will be used for article retrieval.

### Data collection

#### Selection of studies

Two reviewers (MC and JMK) will independently screen the study eligibility and extract data. This will be a two-step process with title and abstract screening followed by full text screening. Keywords used for abstract screening can be seen in Supplementary File 3. Disagreements between the two reviewers will be resolved via consensus or third-party reviewer (TW). Any reports that are duplicates or co-publication will be identified at the screening stage. Excluded studies with the reason for their exclusion will be provided in an appendix of the final report.

#### Data extraction and management

Two researchers (MC and JMK) will develop a data extraction form and this will be piloted initially, with 10% of papers, to achieve a good level of agreement between the researchers. The following data will be extracted separately and in duplicate.

- Study characteristics*:* authors, year of publication, country, design, sample size, clinical setting, number studied, number of drop-outs with reason and funding source
- Population characteristics inclusion/exclusion criteria: demographics such as age and gender. Studies including participants aged > 18 years will be excluded unless data for those < 18 can be separated.
- MR-proADM levels*:* time of sampling, method of sampling
- Gold standard*:* sterile site bacterial culture (blood/CSF) or quantitative PCR
- Outcomes*:* From this 2 × 2 table, we will calculate true positives, false positives, true negatives, and false negatives.

If the two researchers feel that a potential study could be included but may need clarification from the original author or additional data is required a formal process of contacting, the corresponding author will be completed. If the paper has an email address linked to the publication, an email will be sent requesting the required information. If no response is returned within 1 week then a reminder email will be sent. If there is no response 1 week after the reminder email, the researchers will come to an agreement of whether the study can be included minus the additional data requested. If studies do not have an email address linked to the publication and were published after 2005, a reasonable online search for contact details of the corresponding author will be completed to follow the same contact procedure. If contact information is not available or the paper has no contact details and was published before 2005, the respective study will be excluded.

#### Assessment of methodological quality and bias

Bias will be evaluated independently by two researchers (MC and TW) and reported according to the quality assessment of diagnostic accuracy studies (QUADAS-2) tool [27]. This internationally recognised tool provides a structured and standardised way to evaluate the risk of bias and applicability of diagnostic test accuracy research across four domains: (i) patient selection, (ii) index test, (iii) reference standard and (iv) flow and timing. Disagreements between the two researchers will be resolved by consensus or via a third party (MDS). An assessment of publication bias will not be performed in this systematic review. This is based on the recommendations from the Cochrane Collaboration who have called for further research to “improve our understanding of the determinants and extent of publication bias for diagnostic studies”. Current methods for the assessment of publication bias have been developed for use in systematic reviews of randomised control trials (RCTs) for medical interventions. The inherent differences in study design, study size and outcome measures mean that even the reported “best” methods of detecting publication bias have a “low power” and are unreliable [28].

#### Statistical analyses and evidence synthesis

We will present an overview of the available studies summarised in two tables. The first table will summarise the study designs, participants, index tests details, sample types and the reference standards (positive blood, urine or CSF culture). The second table will summarise details on study quality relating to QUADAS-2.

MR-proADM test result data will be compared to the reference test. Data for 2 × 2 tables of index test against reference standard will be extracted from each study. The true positive, true negative, false positive and false negative rate will be recorded. If these data are not provided, they will be calculated from raw data wherever possible.

For each study, we will calculate sensitivities and specificities and their 95% confidence intervals for each reported cutoff. These data will be presented graphically via forest plot with 95% confidence intervals and also in a receiver operating characteristic (ROC) space, this will allow for visual inspection of any between-study variability.

For the main analysis, we will use a common threshold (if it exists). A common threshold describes when all of the studies include the same cutoff value or threshold for predicting the diseased state. We will only pool studies if they are conducted in similar settings and are deemed applicable and at low risk of bias based on both the Standards for Reporting Diagnostic accuracy studies (STARD) and QUADAS-2 tools. For the meta-analysis, we will fit a summary ROC curve using a bivariate random-effects model as described by the Cochrane Collaboration [29]. We will derive summary accuracy statistics (sensitivity and specificity) and plot the 95% confidence interval and prediction region in the ROC space. Analysis will be undertaken using the Stata/IC software version 15 (StataCorp, College Station, Texas) and Review Manager version 5.3.

#### Investigations of heterogeneity

An initial assessment of heterogeneity will be performed via visual inspection of forest plots and of individual study results in the ROC space. Potential sources of heterogeneity include the following:

- Publication year
- Country

##### Reference standard i.e. blood culture, PCR or both

- Age of child i.e. neonate, preschool children and older children

Where possible we will explore the impact of covariates by conducting subgroup analyses in Review Manager 5.3 and by including covariates in the random effects model.

## Discussion

Mid-regional pro-adrenomedullin is a relatively new biomarker that appears to have the key physiological features of an ideal biomarker; in that, it is stable in bodily fluids, easy to measure and has a long half-life. It remains unclear; however, how reliable this biomarker is in predicting invasive infection in children or where in the diagnostic pathway to apply this test. The planned systematic review is important because it will help to summarise the existing literature, identify gaps in our knowledge, report on the available accuracy data and direct future research. This is especially important in children as invasive bacterial infections are notoriously difficult to identify during the prodrome.

As with any new biomarker, the existing data may be limited in volume and quality. This may make meta-analysis difficult and it may not be possible to fully define the role of MR-proADM in the assessment of children with suspected infection. The lack of high quality data may also make subgroup analysis difficult. In those scenarios however, this review will provide a useful guide for researchers to identify gaps in the existing literature and to decide upon future research priorities.

## Conclusion

Invasive bacterial infections are a significant cause of morbidity and mortality in the paediatric population. The early recognition of IBI remains a challenge with a realisation that traditional biomarkers of IBI such as CRP are unreliable. Novel biomarkers such as MR-proADM may represent a significant advancement in the diagnosis of IBI in children. The purpose of this review is to accurately describe the diagnostic accuracy of MR-proADM in diagnosing IBI in children.

It is our intention that this review will evaluate if MR-proADM is suitable for inclusion in the diagnostic pathway of serious IBI in children. We also hope it may highlight areas where evidence is lacking in order to guide future work into this novel biomarker for infections within the paediatric population.

## Supplementary information

**Additional file 1.** PRISMA-P Checklist

**Additional file 2.** Example search strategy

**Additional file 3.** Keywords for abstract screening

## Data Availability

not applicable

## Abbreviations

ADM: Adrenomedullin
AUC: Area under the curve
CL: Confidence limit
CRP: C-reactive protein
CSF: Cerebral spinal fluid
HSROC: Hierarchal summary receiver operator curve
IBI: Invasive bacterial infection
IL-6: Interleukin 6
MR-proADM: Mid-regional pro-adrenomedullin
PCR: Polymerase chain reaction
PCT: Procalcitonin
RCT: Randomised control trial
RR: Relative risk
SROC: Summary receiver operator curve
