# Supplementary material for: A protocol for a systematic review and meta-analysis of the diagnostic accuracy of mid-regional pro-adrenomedullin in predicting invasive bacterial infection in children
Source: Syst Rev. 2020 Apr 2;9:69. doi: 10.1186/s13643-020-01338-1 (PMC7119004; doi:10.1186/s13643-020-01338-1)
Supplement: Supplementary file 3 — Additional file 3. Keywords for abstract screening [file 13643_2020_1338_MOESM3_ESM.docx]

**Keywords for abstract screening**

Words Related to MR-proADM

MR-proADM

Adrenomedullin

Mid-regional pro-adrenomedullin

proADM

Calcitonin-gene related peptide

Words related to target population

Children

Neonates

Infants

Babies

Newborns

Adolescents

Paediatric

Minors

<18 years old

Words related to target condition

Sepsis

Septic Shock

Bacteraemia

Invasive bacterial infection

Serious bacterial infection

Culture positive infection

Meninigism

Meningitis

Bacterial meningitis

Meningococcal Disease

Meningococcal septicaemia

Urosepsis

Urinary tract infection

CSF infection

Synovial fluid infection
